# Molecular Characterization of Helicase and Nuclease Domains in PPV5 NS1 from Mexican Full-Length Sequence

**DOI:** 10.3390/v18060631

**Published:** 2026-05-30

**Authors:** Diana Michele Araiza-Hernández, Alejandro Vargas-Ruiz, Ernesto Marín-Flamand, Rosa Elena Sarmiento-Silva, José Iván Sánchez-Betancourt, Juan Omar Hernández-Ramírez, Lucía Angélica García-Camacho

**Affiliations:** 1 Master’s and Doctoral Program in Animal Production and Health Sciences, Cuautitlán College of Superior Studies, National Autonomous University of Mexico (UNAM), Carretera Cuautitlán-Teoloyucan Km 2.5, Cuautitlán Izcalli 54714, Estado de México, Mexico; sais.bid@hotmail.com; 2 Department of Biological Sciences, Cuautitlán College of Superior Studies, National Autonomous University of Mexico (UNAM), Carretera Cuautitlán-Teoloyucan Km 2.5, Cuautitlán Izcalli 54714, Estado de México, Mexico; patologiavargas30@gmail.com (A.V.-R.); marflamvz@comunidad.unam.mx (E.M.-F.); mvzjohr@cuautitlan.unam.mx (J.O.H.-R.); 3 Virology Laboratory, Department of Microbiology and Immunology, College of Veterinary Medicine and Animal Science, National Autonomous University of Mexico (UNAM), Av. Universidad 3000, Coyoacán, Mexico City 04510, Mexico; rosass@unam.mx; 4 Department of Swine Medicine and Animal Science, College of Veterinary Medicine and Animal Science, National Autonomous University of Mexico (UNAM), Av. Universidad 3000, Coyoacán, Mexico City 04510, Mexico; ivan.sanchez@posgrado.unam.mx

**Keywords:** PPV5, NS1, helicase, nuclease, electrostatic potential, SASA, modeling

## Abstract

PPV5 NS1 is a nonstructural and multifunctional protein comprising helicase and nuclease domains. The helicase domain contains conserved motifs from superfamily 3 helicases, including Walker A, Walker B, Motif B’, Motif C, and Box VII, whereas the nuclease domain consists of glutamate, a HUH motif, lysine, and tyrosine. In Mexico, the reported prevalence of PPV5 is higher than in other countries, with notable amino acid differences compared with pathogenic PPVs. This study compares the helicase and nuclease domains from a full-length PPV5 NS1 sequence with porcine parvovirus 1 (PPV1) and canine parvovirus (CPV) to characterize the protein further and perform three-dimensional (3D) modeling using bioinformatic tools, including solvent-accessible surface area (SASA) and electrostatic potential assessments. The main findings highlight the ATP-binding pocket, showing electrostatic values in PPV5 that contrast with PPV1 and CPV. The electrostatic potential 3D models suggest those differences involve non-conserved regions. In particular, the PPV5 Box VII surface is predominantly negative due to a glutamate substitution at position 7. In the nuclease domain, the interaction with Mg^2+^ differs between PPV5 and pathogenic PPV. The electrostatic findings suggest that these differences may have functional implications for both domains, but confirmation must be completed with functional assays.

## 1. Introduction

Porcine parvovirus 1 (PPV1) is the only recognized pathogenic parvovirus in the swine population. However, emerging porcine parvovirus species (PPV2–PPV8) have been associated with disease [1,2,3,4], although their pathogenic potential remains uncertain. Parvoviruses are small, non-enveloped DNA viruses with a single linear genome that belong to the *Parvoviridae* family. Two main large open reading frames (ORFs) have been identified: ORF1 encodes the nonstructural proteins NS1, NS2, and NS3, while ORF2 encodes the structural proteins VP1, VP2, and VP3 [5,6,7,8]. The NS1 protein is highly conserved [9] among family members and plays essential roles in viral replication, including helicase, ATPase, nuclease, and DNA-binding activities, as well as cytotoxic activity [6,10,11]. The loss of functional domains can disrupt these critical biological functions [6]. The helicase domain, belonging to superfamily 3 (SF3), is encoded by small DNA and RNA viruses. SF3 helicases form hexamers or double hexamers containing four conserved motifs within a 100-amino acid stretch [12]. These motifs include the Walker A motif (GPATTGKT), which features a glycine-rich loop structure that interacts with the phosphate group of ATP; the Walker B motif (VIWWEE); the B′ motif (a 14-amino acid region); the C motif, which contains an invariant asparagine [13]; and Box VII, where the arginine finger (AF) is located. The AF acts as a biomotor that assists in hexamer assembly and participates in the sequential coordination between subunits within the ATPase activity pocket [14,15].

In addition to the helicase motif, the NS1 protein contains a nuclease domain that belongs to the HUH endonuclease superfamily, comprising three groups: conjugate plasmid transfer proteins (relaxases), mobilization proteins (Mob proteins), and rolling circle replication proteins (Rep proteins) [16]. Rep proteins are involved in rolling circle, and rolling-fork replication [17] is the type of replication carried out by PPVs [18]. The nuclease domain is also referred to as the origin-binding domain (OBD) [6,19] because it recognizes the origin of replication (ORI) site in the viral sequence and cleaves the double-stranded (ds) DNA genome [20,21]. Essential amino acids for this cleavage include two histidine residues separated by a hydrophobic amino acid (U), forming the HUH motif; a glutamate (E), which serves as the metal ion coordination site; and a nearby tyrosine (Y), which covalently binds to the cleaved DNA [22].

Porcine parvovirus 5 (PPV5) has two ORFs: ORF1 encodes the 601-amino acid NS1 protein from nucleotide 862 to 2667, and ORF2, which encodes the 991-amino acid VP1 protein from nucleotide 2787 to 5762 [7]. First identified in the United States in 2013, PPV5 has since been reported in China, Poland, Italy, South Korea, Colombia, Brazil, and other countries [23,24,25,26,27].

Mexico, in a retrospective study, revealed an overall prevalence of PPV5 at 32.4% (50/170), with a prevalence of 52% (26/50) in cases of postweaning multisystemic wasting syndrome (PMWS). Both rates are higher than those reported in other countries. Furthermore, a significant relationship between PPV5 and PMWS cases was found [28]. Additionally, the PPV5 helicase motifs exhibited distinct amino acid substitutions near the active sites of functional motifs compared to recognized pathogenic PPVs, potentially altering helicase function. The first three-dimensional (3D) model of the PPV5 helicase domain was also created [29]. In the current study, the solvent-accessible surface area (SASA) of PPV5 helicase motifs was assessed, and 3D models based on the electrostatic potential of the PPV5 helicase domain were generated to further investigate the previously reported substitutions. Given that the pathogenic potential of novel PPVs is currently unknown [30], bioinformatic-based analysis of their proteins may aid in studying their role in disease.

## 2. Materials and Methods

### 2.1. Case Selection

The SASA assessment, electrostatic potential 3D models, probability of ATP binding of the helicase domain, and the predicted amino acid sequences in the nuclease domain analysis along 3D models were completed using the PPV5 Mexican partial ORF1 reported sequences (OQ792211, OQ792212, and OQ792213), which comprise 96.9% of the NS1 protein [28,29], a recently amplified full-length ORF1 sequence (PZ016994). The DNA used to amplify the PPV5 Mexican sequences was extracted from formalin-fixed, paraffin-embedded lymph node tissues derived from PMWS cases in 2005. For comparative analysis with pathogenic virus sequences, the PPV1 (U44978) and canine parvovirus (CPV) (M19296) sequences published in GenBank were utilized.

### 2.2. DNA Extraction from Paraffin-Embedded Tissues

A commercial kit (QIAamp DNA FFPE Tissue Kit, Qiagen, Hilden, Germany) was employed to extract DNA from tissues. The DNA was then amplified by PCR according to the manufacturer’s instructions. DNA quantification was performed using spectrophotometry with a NanoDrop Lite (Thermo Fisher Scientific, Waltham, MA, USA), and 50 ng/µL aliquots were prepared for subsequent storage at −80 °C.

### 2.3. Primer Design

The software programs BioEdit [31] and Primer3 input (v0.4.0, Institute for Biomedical Research, Boston, MA, USA) [32] were used for primer design. Fifteen PPV5 sequences from different countries available in GenBank (www.ncbi.nlm.nih.gov/genbank accessed on 5 February 2021) were compared. The amplification strategy to obtain the complete ORF1 sequence of PPV5 involved designing pairs of first- and second-reaction primers for the amplification of four overlapping fragments via nested PCR (Table 1).

### 2.4. Nested PCR

Genomic DNA (50 µL) from the selected sample was amplified using 2× Master Mix (Ampliqon, Odense, Denmark), which contained 2.0 mM Taq polymerase, 1.5 mM MgCl_2_ 0.4 µM of each dNTP, 10 µM of each primer, and 50 ng of template for the initial reaction. Subsequently, 5 µL of this reaction was used for the nested reaction in a thermal cycler (Mastercycler Gradient, Eppendorf, Hamburg, Germany). The amplification conditions were as follows: an initial denaturation step at 95 °C for 2 min, followed by 40 cycles of denaturation at 94 °C for 35 s, an annealing step at 58 and 60 °C for 35 s, and an extension step at 72 °C for 1 min. A final extension step was performed at 72 °C for 7 min. The products were visualized by electrophoresis in a 1.5% agarose gel stained with ethidium bromide using a UV transilluminator (Apollo Instrumentation, Claremont, CA, USA).

### 2.5. Sequencing

Each amplified product was recovered and purified from the agarose gel using a commercial kit (MinElute Gel Extraction Kit, Qiagen), following the manufacturer’s instructions. The products were then sequenced using a Big Dye Terminator V3.1 kit (Applied Biosystems, Waltham, MA, USA) on a 3500 Genetic Analyzer (Applied Biosystems) at the Institute of Cell Physiology of the National Autonomous University of Mexico.

### 2.6. Molecular Analysis

BioEdit software (v7.2.5, Ibis Bioscience, Carlsbad, CA, USA) [31] was used for sequence editing, assembly, and alignment to obtain the linear ORF1 sequences, as well as for amino acid prediction. Molecular modeling analysis was performed using the Swiss Model program (https://swissmodel.expasy.org/ accessed on 12 May 2023). Given that PPV5 and AAV belong to the *Parvoviridae* family and SF3 helicase is well-preserved among members of this family, comparative modeling is reliable despite divergences within the sequences using available templates. Therefore, the helicase domain 3D models are based on the structure of a hexamer-type Rep protein of an AAV (7jsi.1C model) obtained by electron microscopy, while the nuclease domain 3D models are based on a monomeric type Rep protein of AAV2 (5dcx.4) obtained through X-ray diffraction. Model visualization, editing, and electrostatic potential calculations were completed in PyMOL version 4.6.0). The SASA analysis was performed using the GETAREA program for each functional motif from the helicase domain, comparing the PPV5 Mexican sequences (OQ792211, OQ792212, OQ792213, and PZ016994) and the full-length PPV5 sequence (PZ016994) with pathogenic PPV1 (U44978) and CPV (M19296) sequences. The probability of DNA binding was calculated using ATPbind site prediction from the National University of Singapore. To investigate selection pressures, 19 PPV5 sequences, including the Mexican ones, were analyzed using a codon algorithm based on Z-test selection, comparing the rates of synonymous and non-synonymous substitutions and using 1000 bootstrap replicates, with a syn-non-synonymous substitution model using the Kumar method (Kimura 2-parameter). A positive selection hypothesis (d_N > d_S) was proposed to determine if base mutations that change amino acids are favored by viral evolution.

## 3. Results

### 3.1. Analysis of the Solvent-Accessible Surface Area (SASA) of Helicase

The amplified products from fragments 1 through 4 were recovered for the PZ016994 sequence. Their linear sequence revealed a size of 1794 nucleotides, which correspond to 100% of the NS1 protein (598 amino acids). The SASA analysis of the PPV5 Mexican sequences revealed similar findings, as OQ792212 and OQ792213 are identical [29], as are OQ792211 and PZ016994. Therefore, the full-length PPV5 sequence (PZ016994) was used in the comparative analysis hereafter. It was found that the Walker A (Table 2) and Walker B motifs (Table 3) have positive and negative net charges, respectively; however, these charges are lower in PPV5.

In contrast, the B′ motif (Table 4) exhibits a positive net charge in all three viruses, with the highest charge observed in PPV5 at 347.97. The C motif (Table 5) has no charge in PPV5, while PPV1 and CPV have negative charges of −124.49 and −118.78, respectively.

In the Box VII motif, the Mexican sequence displays a negative net charge (−91.19), while the PPV1 and CPV sequences show positive charges (Table 6).

### 3.2. Electrostatic Potential of Helicase Domain Motifs

**Figure 1:**
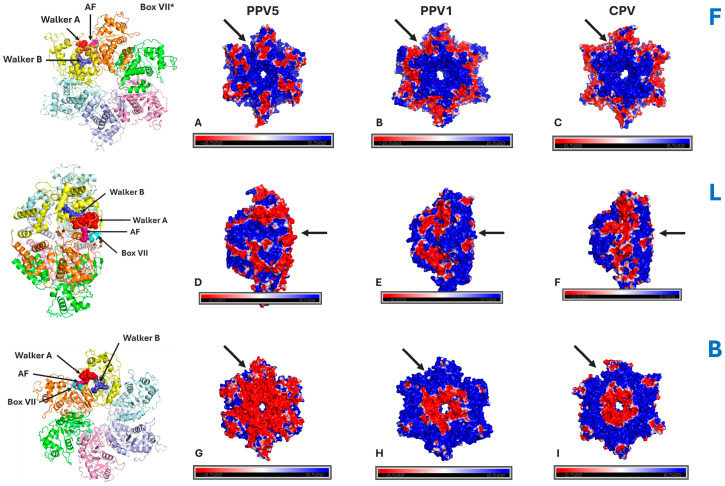
3D models of Helicase Hexamers from PPV5, PPV1, and CPV sequences. The far-left shows models displaying the location of the amino acids (spheres) included in the ATP-binding pocket motifs: Walker A in red, Walker B in blue, Box VII in turquoise, and the arginine finger (AF) in bright pink. * Indicates Box VII is not visible in the first model in the left. Electrostatic potential is shown from the front (**A**–**C**), side (**D**–**F**), and rear (**G**–**I**) views. Positive charges are indicated in blue, and negative charges are indicated in red. Black arrows indicate the junctions between subunits, showing the location of the ATP-binding pocket. The light blue letters indicate: F = front, L = lateral, and B = back.

3D models based on the electrostatic potential for the Mexican PPV5 sequences were created, showing similar illustrations with slight variations due to their molecular identity. The hexameric structure of the NS1 helicase domain is depicted in Figure 1. To visualize the location of functional motifs, 3D models using spheres are provided. The front view reveals that the central region of all three sequences is predominantly positive (shown in blue), although the surface area is smaller in PPV5 than in PPV1 and CPV (Figure 1A, 1B, and 1C, respectively). Similarly, the surface near the junctions with the ATP-binding pocket (black arrows) is positive in PPV5, while the peaks of the hexamer are predominantly negative (red) (Figure 1A). As seen in the side view (Figure 1D), the negative charge extends from the front to the back, covering most of the rear surface (Figure 1G) and the junction between subunits (black arrows). The opposite pattern is observed in PPV1 and CPV, where a negative charge predominates at the junctions between subunits (black arrows) and at the periphery of the hexamer (Figure 1B,C). However, the side views (Figure 1E and 1F, respectively) reveal that this charge is only superficial. Toward the posterior view, the area is predominantly positive (Figure 1H,I), including the surface of the ATP-binding pocket (black arrows). In PPV1 and CPV, only a small area exhibits a negative charge (the central part). As seen in all model views, the central pore has a positive charge (Figure 1E,F,H,I).

**Figure 2:**
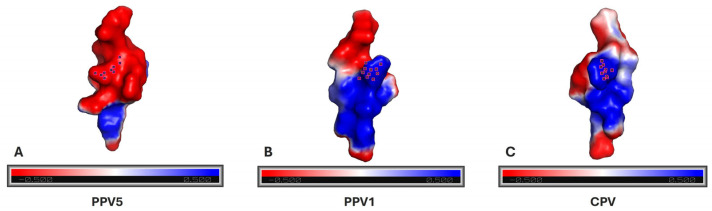
Electrostatic potential of Box VII in the helicase domain of PPV5, PPV1, and CPV. (**A**) Blue dots indicate the surface corresponding to the glutamate (E), while red dots (**B**,**C**) indicate the arginine (R) area.

**Figure 3:**
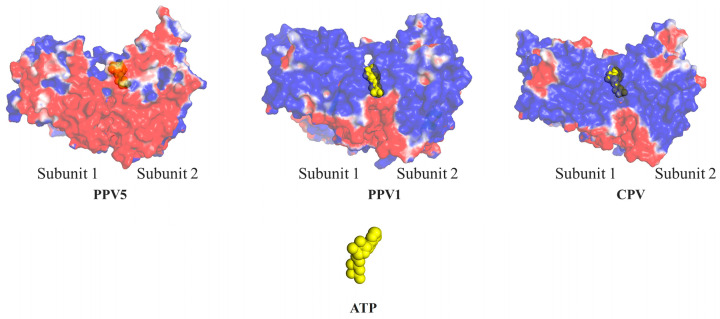
Electrostatic potential of the ATP-binding pocket in the Helicase Domain of PPV5, PPV1, and CPV. Positive charges are indicated in blue, and negative charges are indicated in red. The models are shown in 40% transparency to visualize the ATP molecule (yellow spheres) inside the cleft.

Because the SASA analysis revealed a net negative charge in PPV5 due to the presence of a glutamate residue at position 7, electrostatic potential models were constructed for the Box VII motif. The PPV5 model (Figure 2A) displays extensive negative charge in the vicinity of the glutamate location (blue dots). In contrast, PPV1 and CPV exhibit a positively charged arginine residue at that site (Figure 2B and 2C, respectively), with a predominance of positive charge in the surroundings. Lastly, electrostatic 3D models depicting the ATP binding pocket were created, integrating an ATP molecule in the cleft (Figure 3). A predominance of negative charge at the ATP binding pocket is clearly observed in the PPV5 model, where the ATP is barely visible and deeply inserted in a cleft with indistinct boundaries. Conversely, the pockets in PPV1 and CPV are extensively positive in charge, displaying ATP molecules inserted in clefts with well-defined margins. These features are more evident in PPV1.

### 3.3. Probability of ATP Binding

The probability of ATP binding in the Walker A motif for all the parvovirus sequences revealed similar values, with slightly higher binding observed in PPV1. This binding was noted in all amino acids composing the motif, excluding the first two positions. The Mexican PPV5 sequences exhibited consistent probabilities across each residue position, ranging from 0.489472 to 0.589617, while CPV and PPV1 ranged from 0.500432 to 0.606933 and 0.518261 to 0.654327, respectively (Appendix A Table A1). Additionally, the worldwide PPV sequences used in the previous report (28) exhibited binding probabilities ranging from 0.38724 to 0.561652.

### 3.4. Selection Pressures in the Helicase Domain

The codon-based Z-test used to evaluate natural selection and evolutionary pressure among the sequences obtained under a positive selection hypothesis indicates that mutations are not favored by viral evolution. The Z-values obtained from the Mexican sequences compared to the other sequences are (Z < −1.96) (Appendix A Table A2), suggesting a higher substitution rate without impacting amino acid changes. It is likely that the mutations in the Mexican viruses occurred randomly and do not demonstrate selective pressure. Since the analysis did not indicate positive evolutionary selection in the entire sequences, including functional regions, the analysis based on functional helicase and nuclease regions was not completed.

### 3.5. Amino Acid Prediction for the Nuclease Domain

**Figure 4:**
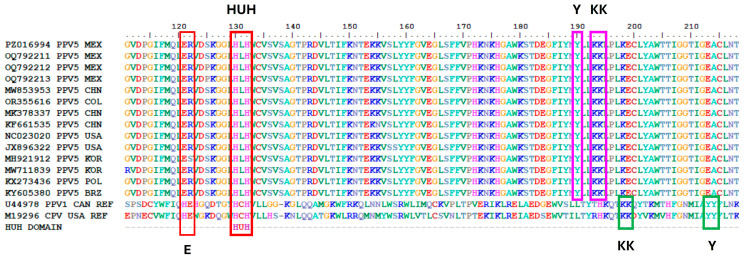
Conserved amino acids in the nuclease domain. Red boxes indicate the amino acids that are most closely conserved between sequences E and HUH. Pink boxes indicate the amino acids from PPV5 (KK and Y), and green boxes indicate the amino acids from PPV1 and CPV (KK and Y) (Graphic view, BioEdit).

The molecular analysis of amino acid prediction in the nuclease functional domain is shown in Figure 4. As mentioned, this comparative analysis included the Mexican full-length sequence (PZ016994), the three previously reported Mexican partial sequences (OQ792211, OQ792212, and OQ792213), which comprise 96.9% of the NS1 protein [29], and the reported PPV1 (U44978) and CPV (M19296) sequences. The four Mexican sequences exhibit amino acids essential for nuclease function: a glutamate (E) residue at position 105; a leucine (L) between the histidines (H) at the HUH motif (positions 114–116); two lysine residues (KK) at positions 177 and 178; and tyrosine (Y) at position 174. PPV1 contains a glutamate (E) at position 120, a cysteine (C) at the HUH motif (positions 128–130), two lysine residues (KK) at positions 195 and 196, and two tyrosines (YY) at positions 210 and 211. CPV has the same amino acids as PPV1 but at different positions 121 (E), 129–131 (HCH), 196–197 (KK), and 211–212 (YY).

### 3.6. Three-Dimensional Model of the NS1 Nuclease Domain

**Figure 5:**
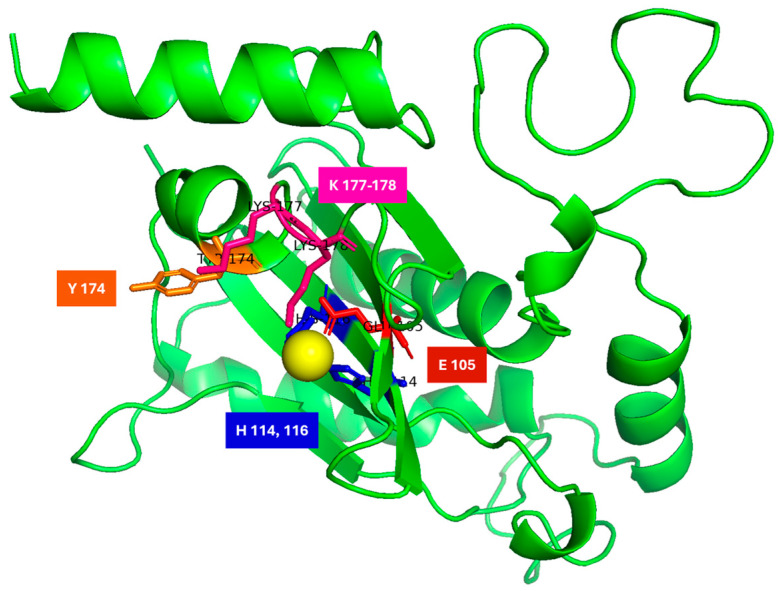
3D model of the PPV5 nuclease domain. Conserved amino acids are colored as follows: red for glutamate (E); blue for histidine (H); pink for lysine (K); and orange for tyrosine (Y). The E, HUH, and K amino acids interact with magnesium (yellow sphere). Ribbon representation of the domain, with important amino acids highlighted in licorice.

**Figure 6:**
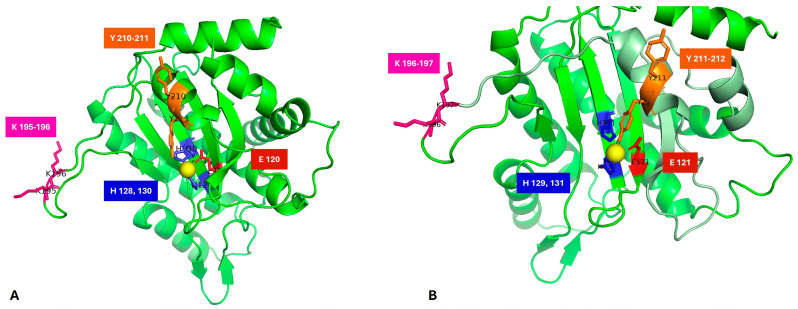
3D model of the nuclease domain of PPV1 (**A**) and CPV (**B**). Conserved amino acids are colored as follows: red for glutamate (E); blue for histidine (H); pink for lysine (K); and orange for tyrosine (Y). The E, HUH, and K amino acids interact with magnesium (yellow sphere). Ribbon representation of the domain, with important amino acids highlighted in licorice.

The 3D model of the nuclease domain, based on the PZ016994 PPV5 sequence (Figure 5), shows that the amino acids located near the conserved region interact with Mg^2+^ (yellow sphere), except for tyrosine (Y) and lysine (K) at positions 174 and 177, respectively. Conversely, the nuclease domain models for PPV1 and CPV (Figure 6) display that glutamate, histidine, and one tyrosine interact with Mg^2+^ (yellow sphere), while the other tyrosine nearby and lysine residues are not in contact with the cation.

## 4. Discussion

The pathogenic potential of novel PPVs has not been fully demonstrated, despite their putative association with well-known viruses causing disease [2,28,33]. Furthermore, it is suggested that they might be commensals due to their high prevalence in healthy animal populations [34]. Unfortunately, isolating and propagating novel PPVs has encountered difficulties, such as slow growth in cell cultures [35] and a high rate of interspecies and common pathogen co-infections [28,36], thus delaying in vitro and in vivo studies that are urgently needed to determine their capacity to cause disease.

While PPV5 was recently isolated and propagated in testicular primary cell cultures from piglets [37], obtaining inoculum for experimental infections and in vitro assays will prove costly and time-consuming. Meanwhile, structural studies of the proteins of novel PPVs using bioinformatic tools, although limited by the lack of correlation with functional assays due to the unavailability of strain repositories, will provide preliminary insights regarding their functions based on what is well-documented in proteins with similar functions and/or related viruses.

NS1 is a multifunctional protein essential for effective viral replication and the production of infectious virions [5,6,14,35]. To fulfill its role, NS1 contains a nuclease domain (DNA-binding domain) and a helicase domain (ATP-binding and hydrolysis domain) [36]. These functions are achieved through electrostatic interactions between the amino acids within the active sites and their ligands [37]. Therefore, in the present work, the NS1 PPV5 structure was investigated based on its electrostatic properties (electrostatic potential and SASA assessment) to aid in understanding the protein’s main activities. To our knowledge, there are no 3D models based on the electrostatic potential of the helicase and nuclease domains, nor SASA assessments in the helicase functional motif of PPV5, PPV1, and CPV.

The amplified full-length Mexican PPV5 sequence (PZ016994) presented the same differences in residues compared to PPV1 and CPV depicted in the three previously described Mexican PPV5 sequences. Such differences were suggested to have functional implications, particularly a substitution of arginine (R) for glutamate (E) at position 7 of Box VII of the helicase domain, where the AF is contained [29]. These substitutions were also observed in worldwide PPV5 sequences.

ATP binding and hydrolysis occur in a cleft known as the ATP-binding pocket. This process involves the interaction between the AF of one subunit and the Walker A and Walker B motifs of the opposite subunit, where direct binding to the phosphates occurs and the essential Mg^2+^ co-factor is provided, respectively. The ATP located in the pocket is susceptible to hydrolysis [38]. This type of activity is known as trans-activation because the AF regulates ATP hydrolysis in the adjacent subunit. Therefore, it is generally accepted that no amino acid alterations are tolerated at these motifs’ active sites [39].

In this context, ATP binding sites exhibit conventional characteristics, such as an abundance of positively charged amino acids and regions that are more accessible to the solvent for promoting ligand interactions. In particular, the Walker A motif in an ATP-binding pocket is enriched with positive residues that interact with phosphate groups. It also features a region that is more accessible to the solvent, facilitating binding with polyphosphate tails [40].

In this study, electrostatic potential models revealed a predominance of negative charge in PPV5, especially throughout the subunit junctions where the ATP-binding pocket is located. Additionally, the PPV5 SASA value was lower in the Walker A and Walker B motifs than in PPV1 and CPV, despite these motifs revealing the same charge in all three species. Taken together, an electrostatic composition with a higher negative charge and smaller solvent-accessible region in PPV5 may hinder ATP binding and/or ATP hydrolysis. In contrast, in PPV1 and CPV, which are recognized as pathogens, the predominance of positive charges and higher SASA values at this particular site may facilitate those functions.

However, the probability of ATP binding reported herein indicates that ATP binding might be achieved in the PPV5 Mexican sequences, which display similar estimates to those obtained for PPV1 and CPV. Such findings may be related to evolutionary conservation since ATP binding in Walker A is preserved despite pathogenic potential [31,41]. Moreover, it was mentioned that the Walker A amino acid substitution reported in the Mexican PPV5 sequences could have no functional effect since it was replaced with a similar residue [29]. Furthermore, mutations in Walker A and Walker B conserved amino acids revealed no effect on ATP binding, but a significant impact on ATP hydrolysis [31,39]. Nevertheless, the latter requires trans-activation, which is mediated by the AF located in the Box VII motif.

Despite the AF being present in all parvovirus sequences used for modeling, an extensive negative charge in PPV5 Box VII was observed, consistent with the SASA value. This change is due to the substitution of arginine for glutamate at a position near the AF (R9), which requires positively charged amino acids on either side [42,43,44]. Such findings may have functional implications since these residues are key to ATP hydrolysis, and changes in this region can prevent the formation of infectious viruses [45]. Notably, in CPV, mutations in positively charged amino acids prevented virion production [10], and a point mutation in this region resulted in the minute virus relating to the mice (MVM) genome’s inability to replicate [46].

Taken together, it appears that the electrostatic differences described in the aforementioned motifs might not be associated with ATP binding since it is highly conserved, but rather with ATP hydrolysis. Still, the existence of functional alterations must be confirmed through DNA unwinding, ATP hydrolysis, or DNA binding assays [47,48] to support that notion.

Nevertheless, the electrostatic illustrations shown in Figure 1 and Figure 3 displayed distinct features between PPV5 and pathogenic PPVs. These differences in charge most likely include non-conserved residues since spreading beyond the essential amino acids was observed. It is well-known that helicase enzymatic activity is extremely conserved through evolution, and it is claimed that non-conserved structural elements might be responsible for other activities that may be specific in each helicase [31]. This assumption might be valid for other properties that could be related to pathogenicity, such as cytopathic effects causing cell necrosis and/or apoptosis that favor viral spreading [49]. For instance, in PPV1, the NS1 protein induces cell death and placental tissue damage, leading to reproductive failure. It also induces mitochondrial damage, increases reactive oxygen species (ROS) generation, and damages host DNA [50]. Furthermore, the predominance of negative charge in PPV5 in the subunits comprising the binding pocket appears to modify the cleft conformation compared to PPV1 and CPV. This finding may alter the interaction with ATP, but it must be validated with the functional assays mentioned above.

Because SF3 helicases require ATP for translocation along DNA [44], coordination of all functional motifs is required rather than individual functionality for proper overall enzyme function [31,51]. Mutations affecting the electrostatic environment have been reported to influence thermodynamics at the macromolecular level, resulting in dysfunctional molecules [47].

On the other hand, the PPV5 sequences analyzed in this study exhibit a Rep-type nuclease domain with an invariant glutamate residue (E105) [23]. The PPV5 3D model showed a typical HUH nuclease structure in which the HUH residues are located in a single β-strand. Unlike PPV1 and CPV, which have two tyrosine residues (YY), there is a single tyrosine residue (Y) at position 174, situated near an α-helix, 12.4 Å distant from the Mg^2+^ and the active site. In this regard, the nuclease domain may comprise one or two tyrosine residues, but there is no evidence that the second tyrosine residue is necessary for replication [48]. However, the tyrosine in PPV5 does not interact with Mg^2+^, unlike the models for PPV1 and CPV, in which both tyrosine residues are 5.1 Å close to the active site, with one interacting with Mg^2+^. This was also depicted in models of PPV1, CPV, and MVM reported by other authors [49]. Both the distance and the lack of interaction with the metal ion may affect the function of the nuclease. It is described that DNA cleavage by endonuclease must invariably bind to the metal [50], and it is dependent on the presence of a metal ion in the adeno-associated virus (AAV) [51]. Additionally, for the nucleophilic attack on the free 3′OH phosphate group, tyrosine requires proximity to the active site and the DNA chain [17]. However, it is essential to corroborate this assumption with oligonucleotide excision reactions using fluorescent anisotropy and/or molecular probes [52].

Lastly, the selection pressure analysis revealed neither positive nor purifying selection, which is consistent with our findings since no differences in critical functional amino acids were disclosed between our Mexican PPV5 and worldwide PPV5 sequences. Furthermore, the 3D electrostatic potential models were similar in PPV5 sequences. Moreover, the Mexican PPV5/OQ792211 sequence is identical to the Chinese PPV5/MW853953 sequence [29]. Here, it was also found that the Mexican PPV5 PZ016994 showed 100% homology to those sequences. In a recent study, a Colombian PPV5/OR3556159 showed high homology to the Chinese PPV5/MW853953. Therefore, the models depicted in the present work are representative of the PPV5 species.

## 5. Conclusions

In conclusion, the main findings from analyzing the SASA and electrostatic potential models of the PPV5 sequences evaluated in this study were related to the motifs forming the ATP-binding pocket of the helicase domain, showing distinct electrostatic features between PPV5 sequences and pathogenic PPVs. In contrast, the findings in the nuclease domain were associated with Mg^2+^ interactions. It is likely that these differences may have significant functional implications, but functional assays are needed to validate these premises. Unfortunately, isolating and propagating emerging PPV species has proven difficult, thus considerably limiting the in vitro and in vivo studies of their pathogenic potential.

## Figures and Tables

**Table 1:** Primers for Amplifying PPV5 ORF1. Primers for the first and second reactions. The primers for fragments 1 through 3 were previously described by [29] Vargas-Ruiz et al. (2025). Fragment 4 covers the final portion of ORF1. Fw = forward, Rv = reverse, Fn = forward nested, Rn = reverse nested.

Fragment	Primer	5′-3′ Sequence	Amplicon	Annealing Temperature
**1**	Fw	TTTGTCATTTGCGTTTTGGA	740 bp	58 °C
	Rv	CATGGGAGCAGATAATTCTTCA		
	Fn	AGCAGAACTCCGTCGTTTTC	611 bp	
	Rn	TCCAATTGTAGTCCATGCATAA		
**2**	Fw	GCATGGAAAAGCACAGATGA	726 bp	58 °C
	Rv	TTTCCCTTCTTCCCACCAC		
	Fn	TGAAAGAATGTCTTTATGCATGG	620 bp	
	Rn	AGGAAAATTTGGATTGTTCCAGT		
**3**	Fw	TGCAAAATTCTTACCTGGAACA	721 bp	60 °C
	Rv	TGCAAAATTCTTACCTGGAACA		
	Fn	GGCAATCTGCCATAGCTCA	650 bp	
	Rn	AATTCTCATAAGCCGCAGGA		
**4**	Fw	AGACGTCTTGGATCATTGGT	740 bp	58 °C
	Rv	ACGGCTTCTTCCAAATCAGT		
	Fn	AGACCCTACAAACACGACACA	696 bp	
	Rn	TTCTTCATCCACGGCTTCTT		

**Table 2:** Walker A SASA. Positively charged amino acids are shown in blue.

WALKER A											
SAMPLE	AA								CHARGE		
	G	P	A	T	T	G	K	T	POSITIVE	NEGATIVE	TOTAL
PZ016994 PPV5	6.93	72.18	35.12	110.22	21.70	0.36	4.37	55.13	4.37		4.37
	**AA**										
	**G**	**P**	**A**	**S**	**T**	**G**	**K**	**S**			
U44978 PPV1	0	59.54	66.67	34.62	2.97	0.73	11.18	71.64	11.18		11.18
M19296 CPV	14.62	69.24	40.39	101.9	14.47	7.23	10.46	50.81	10.46		10.46

**Table 3:** Walker B SASA. Negatively charged amino acids are shown in red.

WALKER B									
SAMPLE	AA						CHARGE		
	V	G	W	W	E	E	POSITIVE	NEGATIVE	TOTAL
PZ016994 PPV5	10.21	0.44	53.87	3.62	48.64	33.88		82.52	82.52
	**AA**								
	**L**	**I**	**W**	**I**	**E**	**E**			
U44978 PPV1	2.31	2.29	16.12	5.53	74.4	62.52		136.92	136.92
M19296 CPV	1.96	0.71	6.09	9.29	86.84	69.15		155.99	155.99

**Table 4:** B′ Motif SASA. Positively charged amino acids are shown in blue, and negatively charged amino acids are shown in red.

B′ MOTIF																	
SAMPLE	AA														CHARGE		
	K	A	L	L	G	G	T	A	L	R	I	D	R	K	POSITIVE	NEGATIVE	TOTAL
PZ016994 PPV5	57.95	42.81	10.72	2.08	30.12	27.90	69.48	52.80	69.51	189.84	73.48	93.77	97.95	123.00	468.74	93.77	374.97
	**AA**																
	**K**	**A**	**I**	**C**	**S**	**G**	**Q**	**T**	**I**	**R**	**I**	**D**	**Q**	**K**			
U44978 PPV1	53.17	31.38	6.52	2.7	29.89	0	107	53.39	68.79	182.94	21.75	80.81	25.56	137.83	373.94	80.81	293.13
M19296 CPV	25.39	42.72	16.83	1.9	25.42	23.77	115.01	65.33	38.38	170.19	18.63	92.07	29.97	191.34	386.92	92.07	294.85

**Table 5:** C Motif SASA. Negatively charged amino acids are shown in red.

MOTIVO C										
SAMPLE	AA							CARGA		
	F	I	I	T	S	N	V	POSITIVE	NEGATIVE	TOTAL
PZ016994 PPV5	1.16	4.30	3.64	6.61	2.14	41.68	98.41			
	**AA**									
	**V**	**I**	**M**	**T**	**T**	**N**	E			
U44978 PPV1	0.38	9.52	6.66	12.57	2.26	41.27	124.49		124.49	124.49
M19296 CPV	2.58	14.66	2.12	12.58	0.83	45.69	118.78		118.78	118.78

**Table 6:** Box VII SASA. Positively charged amino acids are shown in blue, and negatively charged amino acids are shown in red.

BOX VII														
SAMPLE	AA											CHARGE		
	E	H	Q	Q	P	L	E	D	R	M	I	POSITIVE	NEGATIVE	TOTAL
PZ016994 PPV5	158.89	115.99	54.86	118.41	55.66	13.18	83.46	83.20	118.37	18.26	30.51	234.36	325.55	91.19
	**AA**													
	**E**	**H**	**T**	**Q**	**P**	**I**	**R**	**D**	**R**	**M**	**L**			
U44978 PPV1	101.87	53.79	18.26	114.86	38.81	19.21	98.7	68.97	57.52	5.57	32.12	210.01	170.84	39.17
M19296 CPV	136.02	64.82	20.12	116.27	46.8	11.95	109.29	52.37	78.72	4.79	39.88	252.83	188.39	64.44

## Data Availability

The original contributions presented in this study are included in the article. Further inquiries can be directed to the corresponding author.

## Abbreviations

The following abbreviations are used in this manuscript:

PPV1	Porcine parvovirus 1
ORF	Open reading frames
NS	Nonstructural proteins
VP	Structural proteins
SF3	Superfamily 3
AF	Arginine finger
Mob proteins	Mobilization proteins
Rep proteins	Replication proteins
OBD	Origin-binding domain
ORI	Origin of replication
Ds	Double stranded
U	Hydrophobic amino acid
E	Glutamate
Y	Tyrosine
PPV5	Porcine parvovirus 5
PMWS	Postweaning multisystemic wasting syndrome
3D	Three-dimensional model
SASA	Solvent-accessible surface area
PPVs	Porcine parvovirus
CPV	Canine parvovirus
PCR	Polymerase chain reaction
Ng	Nanograms
µL	Microliters
°C	Degrees Celsius
Fw	Forward
Rv	Reverse
Fn	Forward nested
Rn	Reverse nested
Bp	Base pairs
Mm	Milligrams
MgCl_2_	Magnesium chloride
µM	Micrograms
dNTP	Deoxynucleotide triphosphates
Min	Minutes
S	Seconds
F	Front
L	Lateral
B	Back
L	Leucine
H	Histidine
K	Lysine
C	Cysteine
R	Arginine
Mg^2+^	Magnesium
MVM	Minute virus of mice
ROS	Reactive oxygen species
AAV	Adeno-associated virus

## Appendix A

**Table A1:** Probability of ATP binding to the Walker A motif in PPV5, PPV1, and CPV helicase domain. N = no binding B = binding.

Sample	Amino Acids	ATP Bind Probability	ATP Binding Site
**PZ016994** **OQ792211** **OQ792212** **OQ792213**	G	0.011037	N
	P	0.093387	N
	A	0.549603	B
	T	0.544364	B
	T	0.489472	B
	G	0.548799	B
	K	0.589617	B
	T	0.523449	B
**U44978 PPV1 CAN**	G	0.011903	N
	P	0.14869	N
	A	0.533223	B
	S	0.518261	B
	T	0.545409	B
	G	0.654327	B
	K	0.634324	B
	S	0.573076	B
**M19296 CPV USA**	G	0.011523	N
	P	0.133064	N
	A	0.500432	B
	S	0.514695	B
	T	0.50248	B
	G	0.581436	B
	K	0.606933	B
	S	0.51898	B

**Table A2:** Selection pressures in PPV5 helicase domain.

STRAIN 1	STRAIN 2	Dist
PZ016994 PPV5 MEX	OQ792211 PPV5 MEX	−0.003290
PZ016994 PPV5 MEX	OQ792212 PPV5 MEX	0.003665
OQ792211 PPV5 MEX	OQ792212 PPV5 MEX	0.000366
PZ016994 PPV5 MEX	OQ792213 PPV5 MEX	−0.008295
OQ792211 PPV5 MEX	OQ792213 PPV5 MEX	−0.011656
OQ792212 PPV5 MEX	OQ792213 PPV5 MEX	−0.011980
PZ016994 PPV5 MEX	KU745628 PPV5 CHN	0.003378
OQ792211 PPV5 MEX	KU745628 PPV5 CHN	0.000083
OQ792212 PPV5 MEX	KU745628 PPV5 CHN	0.007060
OQ792213 PPV5 MEX	KU745628 PPV5 CHN	−0.004907
PZ016994 PPV5 MEX	MN326219 PPV5 CHN	−0.008610
OQ792211 PPV5 MEX	MN326219 PPV5 CHN	−0.008626
OQ792212 PPV5 MEX	MN326219 PPV5 CHN	−0.004958
OQ792213 PPV5 MEX	MN326219 PPV5 CHN	−0.017044
KU745628 PPV5 CHN	MN326219 PPV5 CHN	−0.005233
PZ016994 PPV5 MEX	MK378337 PPV5 CHN	−0.008281
OQ792211 PPV5 MEX	MK378337 PPV5 CHN	−0.004952
OQ792212 PPV5 MEX	MK378337 PPV5 CHN	−0.004637
OQ792213 PPV5 MEX	MK378337 PPV5 CHN	−0.013339
KU745628 PPV5 CHN	MK378337 PPV5 CHN	−0.004933
MN326219 PPV5 CHN	MK378337 PPV5 CHN	−0.013660
PZ016994 PPV5 MEX	MW853953 PPV5 CHN	−0.004952
OQ792211 PPV5 MEX	MW853953 PPV5 CHN	−0.008281
OQ792212 PPV5 MEX	MW853953 PPV5 CHN	−0.001300
OQ792213 PPV5 MEX	MW853953 PPV5 CHN	−0.013367
KU745628 PPV5 CHN	MW853953 PPV5 CHN	−0.000324
MN326219 PPV5 CHN	MW853953 PPV5 CHN	−0.013629
MK378337 PPV5 CHN	MW853953 PPV5 CHN	−0.013343
PZ016994 PPV5 MEX	KF661535 PPV5 CHN	−0.005758
OQ792211 PPV5 MEX	KF661535 PPV5 CHN	−0.009094
OQ792212 PPV5 MEX	KF661535 PPV5 CHN	−0.002057
OQ792213 PPV5 MEX	KF661535 PPV5 CHN	−0.014226
KU745628 PPV5 CHN	KF661535 PPV5 CHN	−0.002320
MN326219 PPV5 CHN	KF661535 PPV5 CHN	−0.014530
MK378337 PPV5 CHN	KF661535 PPV5 CHN	−0.014166
MW853953 PPV5 CHN	KF661535 PPV5 CHN	−0.010812
PZ016994 PPV5 MEX	MG345028 PPV5 CHN	−0.006629
OQ792211 PPV5 MEX	MG345028 PPV5 CHN	−0.003308
OQ792212 PPV5 MEX	MG345028 PPV5 CHN	−0.002980
OQ792213 PPV5 MEX	MG345028 PPV5 CHN	−0.011656
KU745628 PPV5 CHN	MG345028 PPV5 CHN	−0.003261
MN326219 PPV5 CHN	MG345028 PPV5 CHN	−0.012008
MK378337 PPV5 CHN	MG345028 PPV5 CHN	−0.001644
MW853953 PPV5 CHN	MG345028 PPV5 CHN	−0.011682
KF661535 PPV5 CHN	MG345028 PPV5 CHN	−0.012500
PZ016994 PPV5 MEX	MZ577038 PPV5 CHN	−0.007309
OQ792211 PPV5 MEX	MZ577038 PPV5 CHN	−0.010642
OQ792212 PPV5 MEX	MZ577038 PPV5 CHN	−0.003652
OQ792213 PPV5 MEX	MZ577038 PPV5 CHN	−0.015758
KU745628 PPV5 CHN	MZ577038 PPV5 CHN	−0.003930
MN326219 PPV5 CHN	MZ577038 PPV5 CHN	−0.016022
MK378337 PPV5 CHN	MZ577038 PPV5 CHN	−0.015698
MW853953 PPV5 CHN	MZ577038 PPV5 CHN	−0.012333
KF661535 PPV5 CHN	MZ577038 PPV5 CHN	−0.013238
MG345028 PPV5 CHN	MZ577038 PPV5 CHN	−0.014025
PZ016994 PPV5 MEX	NC023020 PPV5 USA	−0.004095
OQ792211 PPV5 MEX	NC023020 PPV5 USA	−0.000774
OQ792212 PPV5 MEX	NC023020 PPV5 USA	−0.000431
OQ792213 PPV5 MEX	NC023020 PPV5 USA	−0.009087
KU745628 PPV5 CHN	NC023020 PPV5 USA	−0.000712
MN326219 PPV5 CHN	NC023020 PPV5 USA	−0.009453
MK378337 PPV5 CHN	NC023020 PPV5 USA	0.000890
MW853953 PPV5 CHN	NC023020 PPV5 USA	−0.009147
KF661535 PPV5 CHN	NC023020 PPV5 USA	−0.009864
MG345028 PPV5 CHN	NC023020 PPV5 USA	0.002534
MZ577038 PPV5 CHN	NC023020 PPV5 USA	−0.011465
PZ016994 PPV5 MEX	MW051674 PPV5 USA	−0.022561
OQ792211 PPV5 MEX	MW051674 PPV5 USA	−0.026024
OQ792212 PPV5 MEX	MW051674 PPV5 USA	−0.018933
OQ792213 PPV5 MEX	MW051674 PPV5 USA	−0.031399
KU745628 PPV5 CHN	MW051674 PPV5 USA	−0.019180
MN326219 PPV5 CHN	MW051674 PPV5 USA	−0.031497
MK378337 PPV5 CHN	MW051674 PPV5 USA	−0.031264
MW853953 PPV5 CHN	MW051674 PPV5 USA	−0.027768
KF661535 PPV5 CHN	MW051674 PPV5 USA	−0.028645
MG345028 PPV5 CHN	MW051674 PPV5 USA	−0.029509
MZ577038 PPV5 CHN	MW051674 PPV5 USA	−0.023249
NC023020 PPV5 USA	MW051674 PPV5 USA	−0.026929
PZ016994 PPV5 MEX	JX896322 PPV5 USA	−0.000916
OQ792211 PPV5 MEX	JX896322 PPV5 USA	−0.004248
OQ792212 PPV5 MEX	JX896322 PPV5 USA	0.002758
OQ792213 PPV5 MEX	JX896322 PPV5 USA	−0.009301
KU745628 PPV5 CHN	JX896322 PPV5 USA	0.002476
MN326219 PPV5 CHN	JX896322 PPV5 USA	−0.009584
MK378337 PPV5 CHN	JX896322 PPV5 USA	−0.009313
MW853953 PPV5 CHN	JX896322 PPV5 USA	−0.005942
KF661535 PPV5 CHN	JX896322 PPV5 USA	−0.004754
MG345028 PPV5 CHN	JX896322 PPV5 USA	−0.007651
MZ577038 PPV5 CHN	JX896322 PPV5 USA	−0.008281
NC023020 PPV5 USA	JX896322 PPV5 USA	−0.005097
MW051674 PPV5 USA	JX896322 PPV5 USA	−0.023707
PZ016994 PPV5 MEX	MH921912 PPV5 KOR	n/c
OQ792211 PPV5 MEX	MH921912 PPV5 KOR	−0.001981
OQ792212 PPV5 MEX	MH921912 PPV5 KOR	n/c
OQ792213 PPV5 MEX	MH921912 PPV5 KOR	−0.007001
KU745628 PPV5 CHN	MH921912 PPV5 KOR	n/c
MN326219 PPV5 CHN	MH921912 PPV5 KOR	−0.007313
MK378337 PPV5 CHN	MH921912 PPV5 KOR	−0.006980
MW853953 PPV5 CHN	MH921912 PPV5 KOR	−0.003645
KF661535 PPV5 CHN	MH921912 PPV5 KOR	−0.004454
MG345028 PPV5 CHN	MH921912 PPV5 KOR	−0.005322
MZ577038 PPV5 CHN	MH921912 PPV5 KOR	−0.006006
NC023020 PPV5 USA	MH921912 PPV5 KOR	−0.002785
MW051674 PPV5 USA	MH921912 PPV5 KOR	−0.021301
JX896322 PPV5 USA	MH921912 PPV5 KOR	0.000396
PZ016994 PPV5 MEX	MW711839 PPV5 KOR	−0.000799
OQ792211 PPV5 MEX	MW711839 PPV5 KOR	−0.004095
OQ792212 PPV5 MEX	MW711839 PPV5 KOR	0.002865
OQ792213 PPV5 MEX	MW711839 PPV5 KOR	−0.009113
KU745628 PPV5 CHN	MW711839 PPV5 KOR	0.002582
MN326219 PPV5 CHN	MW711839 PPV5 KOR	−0.006112
MK378337 PPV5 CHN	MW711839 PPV5 KOR	−0.009091
MW853953 PPV5 CHN	MW711839 PPV5 KOR	−0.005756
KF661535 PPV5 CHN	MW711839 PPV5 KOR	−0.006558
MG345028 PPV5 CHN	MW711839 PPV5 KOR	−0.007431
MZ577038 PPV5 CHN	MW711839 PPV5 KOR	−0.008125
NC023020 PPV5 USA	MW711839 PPV5 KOR	−0.004893
MW051674 PPV5 USA	MW711839 PPV5 KOR	−0.023437
JX896322 PPV5 USA	MW711839 PPV5 KOR	−0.001715
MH921912 PPV5 KOR	MW711839 PPV5 KOR	0.000512
PZ016994 PPV5 MEX	KX273436 PPV5 POL	−0.009685
OQ792211 PPV5 MEX	KX273436 PPV5 POL	−0.006374
OQ792212 PPV5 MEX	KX273436 PPV5 POL	−0.006017
OQ792213 PPV5 MEX	KX273436 PPV5 POL	−0.018147
KU745628 PPV5 CHN	KX273436 PPV5 POL	−0.006287
MN326219 PPV5 CHN	KX273436 PPV5 POL	−0.015088
MK378337 PPV5 CHN	KX273436 PPV5 POL	−0.011357
MW853953 PPV5 CHN	KX273436 PPV5 POL	−0.014722
KF661535 PPV5 CHN	KX273436 PPV5 POL	−0.005939
MG345028 PPV5 CHN	KX273436 PPV5 POL	−0.009705
MZ577038 PPV5 CHN	KX273436 PPV5 POL	−0.017136
NC023020 PPV5 USA	KX273436 PPV5 POL	−0.007117
MW051674 PPV5 USA	KX273436 PPV5 POL	−0.032609
JX896322 PPV5 USA	KX273436 PPV5 POL	−0.011970
MH921912 PPV5 KOR	KX273436 PPV5 POL	−0.008389
MW711839 PPV5 KOR	KX273436 PPV5 POL	−0.010495
PZ016994 PPV5 MEX	KX352458 PPV5 POL	−0.012584
OQ792211 PPV5 MEX	KX352458 PPV5 POL	−0.009267
OQ792212 PPV5 MEX	KX352458 PPV5 POL	−0.008912
OQ792213 PPV5 MEX	KX352458 PPV5 POL	−0.021087
KU745628 PPV5 CHN	KX352458 PPV5 POL	−0.009174
MN326219 PPV5 CHN	KX352458 PPV5 POL	−0.018026
MK378337 PPV5 CHN	KX352458 PPV5 POL	−0.014263
MW853953 PPV5 CHN	KX352458 PPV5 POL	−0.017641
KF661535 PPV5 CHN	KX352458 PPV5 POL	−0.008827
MG345028 PPV5 CHN	KX352458 PPV5 POL	−0.012610
MZ577038 PPV5 CHN	KX352458 PPV5 POL	−0.020081
NC023020 PPV5 USA	KX352458 PPV5 POL	−0.009998
MW051674 PPV5 USA	KX352458 PPV5 POL	−0.035563
JX896322 PPV5 USA	KX352458 PPV5 POL	−0.014870
MH921912 PPV5 KOR	KX352458 PPV5 POL	−0.011295
MW711839 PPV5 KOR	KX352458 PPV5 POL	−0.013393
KX273436 PPV5 POL	KX352458 PPV5 POL	−0.002837
PZ016994 PPV5 MEX	KY605380 PPV5 BRZ	−0.033094
OQ792211 PPV5 MEX	KY605380 PPV5 BRZ	−0.036586
OQ792212 PPV5 MEX	KY605380 PPV5 BRZ	−0.025629
OQ792213 PPV5 MEX	KY605380 PPV5 BRZ	−0.038275
KU745628 PPV5 CHN	KY605380 PPV5 BRZ	−0.031371
MN326219 PPV5 CHN	KY605380 PPV5 BRZ	−0.042213
MK378337 PPV5 CHN	KY605380 PPV5 BRZ	−0.041859
MW853953 PPV5 CHN	KY605380 PPV5 BRZ	−0.038373
KF661535 PPV5 CHN	KY605380 PPV5 BRZ	−0.039616
MG345028 PPV5 CHN	KY605380 PPV5 BRZ	−0.040078
MZ577038 PPV5 CHN	KY605380 PPV5 BRZ	−0.042220
NC023020 PPV5 USA	KY605380 PPV5 BRZ	−0.037349
MW051674 PPV5 USA	KY605380 PPV5 BRZ	−0.042686
JX896322 PPV5 USA	KY605380 PPV5 BRZ	−0.034186
MH921912 PPV5 KOR	KY605380 PPV5 BRZ	−0.031854
MW711839 PPV5 KOR	KY605380 PPV5 BRZ	−0.033986
KX273436 PPV5 POL	KY605380 PPV5 BRZ	−0.043463
KX352458 PPV5 POL	KY605380 PPV5 BRZ	−0.046581

n/c denotes cases in which it was not possible to estimate evolutionary distances.
